# Call For Poems In Msm Poems Section On Medicine, Health And Human Behaviour

**DOI:** 10.4103/0973-1229.27615

**Published:** 2006

Interested poets may send their work for consideration. They may be in verse, but preferably in blank verse. A four-line write up on the poet with a photograph must accompany every submission. You may submit not more than three poems at one time.

While there cannot really be a restriction of any nature as to topics in poetry, poems dealing with medicine, health, delicate satire on human behaviour of various types, insighful reflections etc are more likely to be accepted for publication.

Poems may preferably be submitted in Word format by email and addressed to the Editor.

Acceptance, or otherwise, will be conveyed within four weeks of receipt of submission, if not earlier. We do not wish to increase the anguish of poets by making them wait further! That may lead to another distressed poetry because of us!

Poems for potential publication in Mens Sana Monographs Vol IV, No 5, January 2007 should reach the Editor in Microsoft Word format by 1^st^ Oct. 2006. All poems will be submitted for peer review and a decision of acceptance or otherwise will be conveyed to the authors by 1st Nov. 2006.

Authors may contact the Editor, *Mens Sana Monographs*, for further details and clarification (email: *mensanamonographs@yahoo.co.uk*). They may also visit the website

www.msmonographs.org

http://mensanamonographs.tripod.com

for further information.

